# Factors that influence persistence or recurrence of high-grade squamous intraepithelial lesion with positive margins after the loop electrosurgical excision procedure: a retrospective study

**DOI:** 10.1186/s12885-015-1748-1

**Published:** 2015-10-20

**Authors:** Menghan Zhu, Yuan He, Jan PA Baak, Xianrong Zhou, Yuqing Qu, Long Sui, Weiwei Feng, Qing Wang

**Affiliations:** 1 Department of Gynecology, Obstetrics and Gynecology Hospital, Fudan University, Shen Yang Road 128, Shanghai, 200090 China; 2 Shanghai Key Laboratory of Female Reproductive Endocrine-Related Disease, Fudan University, Shanghai, China; 3 Department of Pathology, Obstetrics and Gynecology Hospital, Fudan University, Shanghai, China; 4 Department of Cervical Diseases, Obstetrics and Gynecology Hospital, Fudan University, Shanghai, China

**Keywords:** HSIL, LEEP, Positive margins, Recurrence, Persistence, Follow-up

## Abstract

**Background:**

In 5–20 % of patients with cervical high-grade squamous intraepithelial lesion (HSIL), a positive margin after the loop electrosurgical excision procedure (LEEP) is associated with persistence/recurrence, but the prognostic value of other clinico-pathological factors is less clear.

**Methods:**

Among 4336 patients with HSIL who underwent an initial LEEP, 275 (6 %) had HSIL-positive margins, 37 of whom were lost to follow-up. We evaluated the remaining 238 patients. Persistence/recurrence was defined as histopathological HSIL during follow-up.

**Results:**

The age of the patients ranged from 21 to 69 years (median: 40). The median follow-up period was 25 months (range: 6–43). Of the 238 patients, 211 (88.7 %) patients remained free of persistence/recurrence, while 27 (11.3 %) experienced persistence/recurrence. According to a univariate analysis, age (*P* = 0.03) and maximum specimen diameter (*P* = 0.043) were associated with persistence/recurrence, but number/location of involved margin sections and the pathology of the endocervical curettage were not (*P* > 0.10). The relative risk of the subjects (greater than or equal to 35 years ages) was 4.6 times of the subject less than 35 years, the difference was statistically significant (14 % vs. 3 %, *P* < 0.05). A multivariate analysis indicated that an age of 35 years or older was the only independent risk factor (OR 4.97, 95 % CI 1.14–21.62, *P* = 0.03).

**Conclusion:**

In patients with HSIL and HSIL-involved margins after an initial LEEP, age is a strong independent predictor of persistence/recurrence. Follow-up with screening cytology and/or biopsy may be considered in younger patients, whereas a secondary LEEP/hysterectomy may be considered in older patients.

## Background

Worldwide, cervical cancer is the most common malignant tumor of the female reproductive system. Cervical intraepithelial neoplasia (CIN) 2/3, which is also called high-grade squamous intraepithelial lesion (HSIL) according to the Bethesda system, is a well-defined precursor lesion of cervical invasive squamous cell carcinoma and is much more frequent than its invasive counterpart. Cervical cancer can be prevented by early detection and proper treatment of HSIL. However, there is a trend toward conservative treatment, particularly in young women and/or in those who desire to preserve fertility. As one type of cervical conization surgery, the loop electrosurgical excision procedure (LEEP) has been widely applied with ideal therapeutic effects. However, 2–48 % of patients with HSIL who are treated with LEEP have been reported to have persistent/recurrent disease after an initial LEEP for HSIL [1–7].

Many studies have been conducted that have investigated the predictors of persistent/recurrent HSIL. Age, histological grade of the conization specimen, number of involved margin sections, location of involved margin sections, preoperative human papilloma virus (HPV) load, postoperative HPV status, pathology of the endocervical curettage (ECC), and human immunodeficiency virus (HIV) infection are among the possible predictors of persistent/recurrent HSIL [6–13]. A positive margin after LEEP (defined as a histopathological finding of CIN along the specimen margin regardless of the CIN grade) is a well-defined predictor of persistent/recurrent disease [4, 11, 12, 14]. Some investigations have suggested that secondary conization (including cold knife conization and LEEP) or hysterectomy should be applied in patients who have positive margins, while other studies have demonstrated that this population can be followed-up without the need for secondary surgery.

As the spontaneous regression rate of HSIL is much lower than that of LSIL [15], it is reasonable to assume that patients with HSIL margins are more likely to have persistence/recurrence than patients with LSIL margins; therefore, a “wait-and-see” strategy would carry a high risk for persistence/recurrence in patients with HSIL margins in the initial cervical cone specimen. In contrast, if this hypothesis cannot be validated, secondary surgery for these patients may result in overtreatment to a certain extent. A previous study demonstrated that HSIL can regress [16], which definitely challenged this hypothesis. However, as data on the persistence/recurrence rate in patients with HSIL, LSIL margins or HSIL margins are not available, the optimal treatment for patients with HSIL with positive margins remains controversial.

Therefore, we analyzed the data of patients with HSIL and HSIL margins to distinguish the factors that influence persistent/recurrent disease.

## Methods

This study was approved by the ethics committee of Obstetrics and Gynecology Hospital of Fudan University. We reviewed the medical records of all 6443 patients who had undergone LEEP at the Obstetrics and Gynecology Hospital of Fudan University from October 2010 to September 2013. All of these 6443 patients underwent LEEP due to abnormal cervical biopsy revealed as LSIL or higher.

Of these patients, 4336 were diagnosed with HSIL, 895 were diagnosed with LSIL, 903 were diagnosed with chronic cervicitis, 308 were diagnosed with invasive cervical cancer, and 1 was diagnosed with neuroendocrine carcinoma. Among the 4336 patients with HSIL, 275 (6.34 %) had HSIL-involved surgical margins and the others were margin-free or LSIL-involved margins. In 275 patients, 37 were excluded due to loss of follow-up or rejection of follow-up. Finally, a total of 238 patients were enrolled in this study.

The data in the study were collected from hospital’s archived database and for the purpose of research. This study didn’t involve in the diagnosis and treatment of the patients. Informed consent was waived by the the ethics committee of Obstetrics and Gynecology Hospital of Fudan University.

Information that was collected included patient characteristics, details regarding the initial LEEP specimens (e.g., depth, thickness and maximum diameter), histology of the first LEEP specimen and ECC specimen, number and location of involved margin sections, cervical cytology results, high-risk HPV results, biopsy and ECC findings during follow-up, and histology from the second LEEP and hysterectomy after the initial LEEP.

### The initial LEEP

The initial LEEP was performed in an outpatient setting by attending physicians in department of Cervical Disease of Obstetrics and Gynecology hospital through a standard procedure with a loop electrode attached to an electrosurgical unit. The unit was operated in a blended mode that consisted of 70 W for cutting and 30 W for coagulation. In all of the LEEPs performed, specimens were obtained from the transformation zone and the endocervical canal for histopathological evaluation.

The standardized procedure was as the follows: on the day of operation, every patient underwent a repeat colposcopy prior to LEEP to indentify cervical lesions and transformation zone. 5 % acetic acid and Lugol’s iodine solution were used in sequence to distinguish cervical lesions and active transformation zone. The excision started at 2 o’clock position of the cervix. A rotary cut was performed with an electrode moving clockwise to remove the entire active transformation zone. The presence of lesions were far from the edge of the specimen (≥3 mm). The depth of excision depended on the transformation zone: 1) approximate 1 cm if the squamo-columnar junction (SCJ) was observed; 2) if the SCJ could not be observed, the addition LEEP in the canal was performed (top hat LEEP) that the depth of the specimen was approximate 2.5 cm. Cauterization was applied on the cervical crater and bleeders for hemostasis. The duration of the operation was about 5 min.

The LEEP specimens were marked length-wise with ink and were radially sectioned after the endocervical portion had been marked for orientation. Before fixation, each LEEP specimen was measured to determine its depth, thickness and maximum diameter. The margins of the LEEP specimens were subdivided into the following categories: the upper margin, lower margin, bilateral margin, outer margin, inner margin and stromal margin.

### Sequential treatment

According to ASCCP’s guidelines: “If CIN2, 3 is identified at the margins of an excisional procedure or post-procedure ECC, cytology and ECC at 4–6 month is preferred, but repeat excision is acceptable and hysterectomy is acceptable if re-excision is not feasible”. So we performed either a strict follow-up or a secondary LEEP or hysterectomy according to patients’ conditions and intentions.

1. Forty-three patients underwent a ThinPrep® cytologic test (TCT) with an HPV test at the first follow-up, which was 3–4 months after the initial LEEP. The subsequent follow-ups occurred every 6–12 months. If the TCT showed abnormal cytology, including atypical squamous cells of undetermined significance (ASC-US), LSIL and HSIL, a biopsy was performed. If the TCT was normal and if two consecutive HPV tests were positive, a biopsy was also performed. If HSIL was confirmed by biopsy, a secondary LEEP or hysterectomy was performed. This group was named TCT group.
2. One hundred and six patients underwent a TCT, an HPV test at the first follow-up, and a colposcopically directed biopsy simultenously. If the cytology was abnormal and the biopsy showed LSIL or a less pathological lesion, the TCT was repeated and a biopsy was performed 3–6 months later. If the biopsy showed HSIL, a second LEEP or hysterectomy was performed. If both the TCT and biopsy were normal, the patients were followed every 6–12 months thereafter. This group was named TCT and biopsy group.
3. Twenty-two patients received a secondary LEEP at a median of 48 days (range 12–120 days) after the initial one (LEEP group). After the second LEEP, the patients were regularly followed-up with a TCT and an HPV test.
4. Sixty-seven patients received hysterectomy at a median of 33 days (range 8–250 days) after the initial LEEP (Hysterectomy group) as they had the following reasons : 1) They feared disease persistence/recurrence unless the uterus was removed and they had no reproductive need; 2) Secondary LEEP was technically impossible; 3) They experienced difficulties with a regular follow-up. After the hysterectomy, the patients were followed-up with the TCT and HPV test to detect vaginal lesions.

During the follow-up period, the cervical cytology sample, which simultaneously contained both an ectocervical and an endocervical sample, was obtained with a disposable ThinPrep® brush (Hologic, Marlborough, MA, USA). The sample was then fixed in ThinPrep® PreservCyt® Solution (Hologic, Marlborough, MA, USA).

Cervical samples for the HPV test were collected with the Digene cervical sampler kit (Digene, Gaithersburg, MD, USA). The sample was then stored in a tube that contained Digene Specimen Transport Medium (Digene, Gaithersburg, MD, USA). An HPV test was performed with the HC2 system. The chemiluminescent reaction was analyzed by a luminometer and compared with the relative light units of clinical samples and the positive control containing 1.0 pg/mL. With regard to a relative light unit, a positive control ratio of 1 or more was considered a positive result. All samples were analyzed for the presence of the HR-HPV types (16, 18, 32, 34, 36, 39, 45, 51, 52, 56, 58, 59 and 68).

Colposcopy was performed with 5 % acetic acid and Lugol’s iodine solution to distinguish cervical lesions during colposcopically directed biopsies. If no obvious abnormality was found after staining,biopsies were taken from 3, 6, 9 and 12 o’clock positions of the cervix. Along with biopsy, the ECC specimens were obtained for histopathological evaluation using an endocervical curette.

A subsequent LEEP was performed with the same approach as the initial LEEP. Laparoscopic hysterectomy was performed by experienced chief physicians.

All specimens were evaluated at the Department of Pathology, Obstetrics and Gynecology Hospital of Fudan University. A consensus was reached for all specimens after an independent review of the original diagnoses by two experienced gynecological pathologists.

### Definition of persistence/recurrence

Persistence/recurrence was defined as histopathological HSIL, which was diagnosed from a biopsy or a subsequent surgical (including hysterectomy and LEEP) specimen at any time after the initial LEEP was performed. HSIL (CIN2/3) was diagnosed based on Pathology and Genetic of Tumours of the Breast and Female Genital Organs, World Organization Classification of Tumours (2003) [17].

### Statistics

Analysis of the data was performed with IBM^©^ SPSS® 20 software for Windows (SPSS Inc., Chicago, IL, USA). Fisher’s exact tests, the *t*-test, one-way analysis of variance (ANOVA), and Mann-Whitney tests were utilized to identify factors that were related to the presence of persistent/recurrent disease in the univariate analysis. Fisher’s exact test was used to determine the age group that was associated with a significantly increased risk of disease persistence/recurrence. Results were considered statistically significant if a *P* < 0.05 was obtained. A multivariate logistic regression was used to determine the independent value of the factors found to be significant in the univariate analysis.

## Results

### Patient characteristics

All patients had HSIL-involved margins, and 96.6 % (230/238) of the primary LEEP HSIL lesions were positive for high-risk HPV. Based on the first follow-up or treatment, the patients were divided into four groups. The median follow-up period was 25 months (range: 6–43 months) for the entire population. For each group, the follow-up times were as follows, and no significant differences were observed between the four groups: ① TCT group: 80–540 days (median: 240); ② TCT and biopsy group: 37–967 days (median: 280); ③ LEEP group: time from initial to second LEEP: 12–120 days (median: 48), after second LEEP: 90–974 days, (median: 188) and entire follow-up: 12–1040 days (median: 180); and ④ Hysterectomy group: time from initial LEEP to hysterectomy: 7–250 days (median: 33), after hysterectomy: 30–900 days (median: 171), entire follow-up: 30–914 days (median: 227).

The patient characteristics are shown in Table 1. The age of the patients ranged from 21 to 69 (median: 40) years. The mean age of the patients in the hysterectomy group was higher than that of patients in the other three groups (47.9 ± 8.7 vs. 37.2 ± 8.0). Other factors, including the diameter, depth and thickness of the LEEP specimen and the number of involved margins, were not different between the four groups. However, in the hysterectomy group, more cases had ECC HSIL compared with the other groups.

**Table 1:** Characteristics of four treatment groups

	TCT	TCT + Biopsy	2nd LEEP	Hysterectomy	p
Age (range)	24–57	21–60	23–47	28–69	
Mean	35.6 ± 8.7	37.9 ± 8.0	37.4 ± 5.8	47.9 ± 8.7	<0.001^a^
Median size of LEEP	35	38	38	46	
Diameter (cm,mean)	1.92 ± 0.33	1.96 ± 0.42	2.13 ± 0.73	1.82 ± 0.41	0.0856^a^
Median (range)	1.8 (1–2.5)	1.8 (1.2–3.5)	2.15 (1–3)	1.8 (1–3)	
Thickness (cm, mean)	0.89 ± 0.22	0.93 ± 0.21	0.91 ± 0.45	0.84 ± 0.20	0.1124^a^
Median (range)	0.8 (0.3–1.8)	0.9 (0.6–1.7)	0.85 (0.3–1.8)	0.8 (0.3–1.5)	
Depth (cm, mean)	1.44 ± 0.29	1.38 ± 0.29	1.54 ± 0.38	1.39 ± 0.33	0.4128^a^
Median	1.5 (1–2.2)	1.2 (0.8–2)	1.55 (1–2)	1.2 (0.8–2)	
Number of involved margins					
Single	42	96	17	57	0.0512^b^
Multiple	1	10	5	10	
Endocervical curettage histopathology					
≤ LSIL	37	101	18	50	<0.001^b^
HSIL	6	5	4	17	

Data analysis

^a^ One way ANOVA

^b^ Fisher exact test

### Rate of persistence/recurrence of cervical HSIL

Among the 4336 patients with HSIL, 275 (6.34 %) had HSIL-involved surgical margins, and 37 were excluded due to loss of follow-up or rejection of follow-up . Of the remaining 238 patients with HSIL and HSIL-positive margins, 211 (88.7 %) remained free of persistent/recurrent disease, while 27 (11.3 %) had persistent/recurrent disease. In the TCT group, 4 patients demonstrated cytology ≥ ASC-US, and 2 of these patients were excluded due to negative biopsy results. HSIL was confirmed in two (2/43, 5 %) patients by biopsy; one patient underwent a second LEEP, and the other patient received a hysterectomy. In the TCT and biopsy group, 11 patients demonstrated TCT cytology ≥ ASC-US. The biopsy results showed 3 patients with cervicitis, 4 with LSIL and 4 with HSIL (4/106, 4 %). Of the 4 patients with HSIL, 3 underwent a second LEEP and 1 received a hysterectomy. In the LEEP group, 7 of the 22 (32 %) patients were found to have persistence of HSIL according to the second LEEP specimen. Afterward, recurrence was not detected in any of these patients. In the hysterectomy group, 14 of the 67 (21 %) patients were found to have HSIL persistence according to the hysterectomy specimen. The persistent/recurrent cases were all confirmed within 8–127 days, except 2 cases in the TCT + biopsy group (226 and 522 days).

### Factors that influence the persistence/recurrence of HSIL

We tested whether a correlation existed between persistence/recurrence and various factors. No difference was noted in the depth and thickness of the LEEP specimens, the number of involved margins, the location of the involved margin section, and the pathology of the ECC (Table 2) between patients with and without persistent/recurrent HSIL. However, the mean age was significantly higher in patients with persistence/recurrence (46.44 ± 11.79 years vs. 39.42 ± 8.87 years, *P* < 0.001). Furthermore, the mean maximum diameter of the LEEP specimens was smaller in patients with persistence/recurrence compared with patients without recurrence (1.75 ± 0.55 cm vs. 1.93 ± 0.40 cm, *P* = 0.043).

**Table 2:** The influence factors for persistence/recurrence

		Persistence/recurrence of HSIL (*n* = 27)	No persistence/recurrence (n = 211)	P value
Age (years)		46.44 ± 11.79 (28 ~ 69)	39.42 ± 8.87 (21 ~ 67)	Two-tailed 0.000, single-tailed 0.000^a^
Age	<35	2 (3 %)	60 (97 %)	0.03^c^
	≥35	25 (14 %)	151 (86 %)	
The features of LEEP specimen	maximum diameter (cm, range)	1.75 ± 0.55 (1.00 ~ 3.00)	1.93 ± 0.40 (1.00 ~ 3.50)	0.043^b^
	Thickness (cm, range)	0.90 ± 0.22 (0.30 ~ 1.80)	0.82 ± 0.23 (0.30 ~ 1.20)	0.120^b^
	Depth (cm, range)	1.40 ± 0.33 (0.80 ~ 2.00)	1.41 ± 0.31 (0.80 ~ 2.20)	0.917^b^
Number of involved margin section	Single	21 (9.9 %)	191 (90.1 %)	0.092^c^
	Multiple	6 (23.1 %)	20 (76.9 %)	
Location of involved margin section	Upper	7 (14.9 %)	40 (85.1 %)	0.681^b^
	Lower	5 (7.0 %)	66 (93.0 %)	
	Lateral	7 (10.6 %)	59 (89.4 %)	
	Outer	2 (13.3 %)	13 (86.7 %)	
	Inner	0 (0)	4 (100 %)	
	Stromal	0 (0)	9 (100 %)	
Pathology of ECC	≤LSIL	24 (11.7 %)	182 (88.3 %)	1.000^c^
	≥HSIL	3 (9.4 %)	29 (90.6 %)	

^a^ *t* test

^b^ Mann-Whitney test

^c^ Fisher exact test

**Fig. 1:**
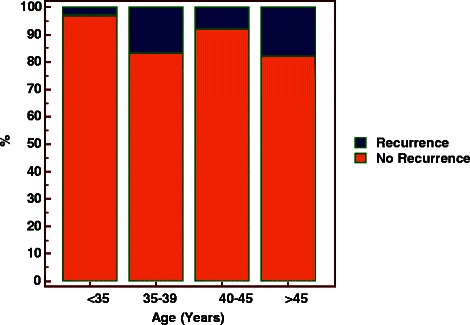
Recurrent rate of patients in four age groups. The rate of patients younger than 35 years was the lowest than that of other groups

**Fig. 2:**
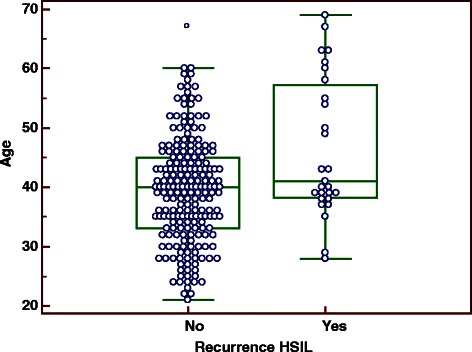
The distributions of age in two groups (recurrent vs. non-recurrent). The distributions of age were different in two groups (recurrent vs. non-recurrent), the median, 25 % percentile and 75 % percentile of age in the recurrent group were greater than the non-recurrent group respectively

**Fig. 3:**
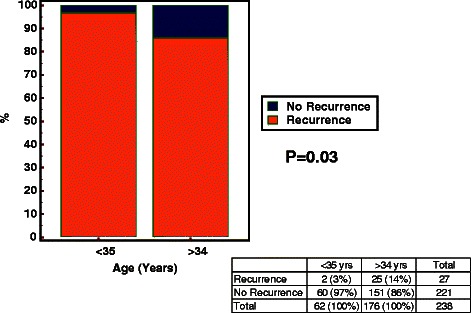
The recurrent rate in two age groups (<35 vs. >34 years). The relative risk of the subjects (greater than or equal to 35 years ages) was 4.6 times of the subject less than 35 years (14 % vs. 3 %, *P* < 0.05)

We further observed the recurrent rate in four age groups. Figure 1 showed the rate of patients younger younger than 35 years was the lowest than that of other groups. In addition, the distributions of age were different in two groups (recurrent vs. non-recurrent), the median, 25 % percentile and 75 % percentile of age in the recurrent group were greater than the non-recurrent group respectively (Fig. 2). In order to clarify the relationship between age and recurrence, these subjects were categorized into two age groups to differentiate the recurrence rate. The relative risk of the subjects (greater than or equal to 35 years ages) was 4.6 times of the subject less than 35 years, the difference was statistically significant (14 % vs. 3 %, *P* < 0.05) (Fig. 3).

A multivariate logistic regression analysis indicated that an age of 35 years or older was the only independent risk factor (OR 4.97, 95 % CI 1.14–21.62, *P* = 0.03).

## Discussion

As both a diagnostic and therapeutic procedure, LEEP provides a conservative approach to treat HSIL, particularly for women who are young or who desire to preserve their fertility. However, cervical lesions persist or recur in a certain portion of patients after LEEP. Positive margins have been identified as a predictive factor of disease persistence/recurrence [4, 11, 12, 14]. As few studies have specifically analyzed the risk of recurrent/persistent HSIL and its related factors in patients with HSIL who have HSIL-involved margins, we specifically investigated the outcome and its related factors in those patients to determine the reasonable treatment options for this population. We found that the rate of persistent/recurrent HSIL was 11.3 % in patients with HSIL with HSIL-involved resection margins at the initial LEEP, as well as that age is a strong independent predictor.

In patients with HSIL, the rate of positive margins after LEEP ranges from 5.7 to 19 % in the literature  [18-23]. In our study, the rate of HSIL margins in patients with HSIL after LEEP was consistent with that in previously published reports (6.34 %). However, in some studies, positive margins are defined as the histological diagnosis of CIN along the LEEP specimen margin, regardless of the CIN grade.

The range of the rates of persistence/recurrence in published references varies greatly, which may be attributed to the different inclusion criteria, definitions of persistence/recurrence and follow-up times. We compared our study with other publications that contained this information as well as predictive factors for persistence/recurrence (Table 3). Among 6 other studies, cases of CIN 2/3 with or without positive margins were included in 5 studies [7, 11, 14, 24-26], whereas cases of CIN1,2 and 3 were included in 1 study [26]. In these studies, the authors also mentioned the rate of persistence/recurrence in patients with positive margins, which varied from 14.2 to 60 %. However, the numbers of patients with positive margins were small (range: 5–71). Furthermore, the definition of a “positive margin” in 4 studies was the presence of CIN (regardless of the grade) [7, 11, 25, 26]. In addition, the definition of recurrence in 4 studies was pathologic findings of CIN3 while in 2 studies was biopsy confirmed evidence of CIN of any grade (1,2,3) [7, 11]. In our study, we found that the persistence/recurrence rate in patients with HSIL with HSIL margins was 11.3 %, which was lower than the rates in the other 6 studies. The possible reason may be our inclusion criteria were critical. Therefore, it remains to be discussed whether subsequent surgeries are necessary for patients who have HSIL-involved margins.

**Table 3:** Rates of persistence/recurrence in patients with positive margin published in literature

Author	Year	Total case No.	Case inclusion criteria	Follow-up period (mean)	No. of cases with positive margin and its definition	Definition of Persistence/Recurrence	Rate of Persistence /Recurrence in cases with positive margin	Predict factor of Persistence/Recurrence^a^
Menghan Z	Current study	238	HSIL with HSIL margin	25 months	238 (the presence of HSIL in the margin)	Histolopathological confirmed HSIL	11.3 % (27/238)	Age and diameter of specimen . in univariate analysis. Age ≥35 was an independent factor
Verguts J [24]	2006	72	CIN 2 or worse	24 months	14 (the presence of CIN	presence of > CIN2, biopsy confirmed	14.2 % (2/14)	Persistence of HR-HPV DNA^a^
Sun X [25]	2009	207	CIN 3	not mentioned	10 (the presence of CIN 1 or worse)^b^	pathologic findings of CIN3	50 % (5/10)^b^	Cytological grade, depth of conization, parity and multi-quadrants of CIN 3 in punch biopsy were related to increased risk for positive margin ^a^
Leguevaque P [7]	2010	352	CIN 2,3	73 months	71 (definition not mentioned)	Pap smear or biopsy confirmed evidence of CIN of any grade (1,2,3)	25.3 % (18/71)	a positive HR-HPV test at 6 months postoperatively,positive endocervical margins,positive pre-treatment HPV typing^a^
Baloglu A [11]	2010	42	CIN 2,3	8.6 months	5 (the presence of CIN 1,2,3)	pathology finding of CIN 1 or CIN 2,3	60 % (3/5)	surgical margin positivity^a^
Kliemann L M [14]	2012	97	CIN 2,3	160 days	39 (the presence of CIN 2,3)	pathology findings of CIN 2,3	46.3 % (18/39)	positive margin, cone height, tumor size^a^
Serati M [26]	2012	282	CIN 1,2,3	26.7 months	21 [the presence of CIN3 was close to (≤1 mm) or involved the margin]	colposcopically directed biopsy proved CIN 3	38.1 %(8/21)	the surgical margin status^a^

^a^The predictive factors were analyzed based on the whole case sample, not the particular subgroup of cases with positive margins

^b^Authors observed residual CIN in 67 of 207 patients who received hysterectomy, but not including those who received other follow-ups

In addition, the authors analyzed the predictive factors in all cases of CIN and found positive margins (3/6 studies), HPV positivity during follow-up (2/6 studies), and tumor characteristics (e.g., depth, height or size) (2/6 studies) were related to recurrence. However, we analyzed the predictive factors in a particular group of patients, namely, those with HSIL and HSIL-involved margins (238 patients), and found that age and diameter of the tumor (size) were associated with persistence/recurrence, while age ≥35 years was the only independent predictive factor.

Increasing age has been identified as a possible pre-surgical predictor of persistence/recurrence in some studies [8, 24, 27]. Our study indicated that in patients with HSIL who have HSIL margins, an age of 35 years or older was an independent risk factor for persistent/recurrent disease. It is still unclear why older women may be more likely to experience persistence/recurrence. One possible reason may be altered immunity or positive selection over time toward viruses with a higher oncogenic risk [24]. In clinical practice, patients in this age group often desire fertility or uterus preservation; thus, conservative management is frequently considered during follow-up. Given the relatively high risk of persistence/recurrence, patients who are older than 35 years require close follow-up.

The size of the lesion is also a factor that has been reported to predict recurrence because it correlates with the completeness of the excision/ablation [28]. The maximum diameter of the LEEP specimen is a reflection of the lesion size. In our study, the maximum diameter was shown to be statistically significant in a univariate analysis. Patients who experienced persistence/recurrence had LEEP specimens with smaller maximum diameters than patients who were free of disease. However, maximum tumor diameter was not an independent risk factor in the multivariate logistic regression. The causes of this difference may be the variation of cervix sizes.

Some studies indicated that extensive involvement of the endocervical cone margin [9] and involvement of multiple margins after LEEP for CIN 2/3 are strong predictors of residual disease. In our study, ECC ≥ HSIL and, multiple involved margin sections were not associated with persistence/recurrence according to a univariate analysis. However, it was notable that the rate of persistence/recurrence was higher in patients with multiple involved margin sections compared with patients who had a single involved margin (23.08 % vs. 9.91 %, *P* = 0.092). Thus,the the significance of involvement of multiple margin sections needs to be investigated in large sample size study.

High-risk HPV (HR-HPV) testing is also useful to predict recurrence. Alonso et al. demonstrated that HR-HPV load (N1000 RLU) prior to LEEP was significantly associated with a higher risk of recurrence, and the most important predictor of recurrence was a positive HR-HPV test at 6 months post-surgery [4]. Another study demonstrated that a test for HPV DNA conducted 12 months post-therapy was the best predictor of recurrent or residual disease [29]. Ovestad et al. found that CIN 2/3 lesions with non-HPV16 HR-HPV infection may spontaneously regress [30]. Since 2006, the guidelines of the American Society for Colposcopy and Cervical Pathology (ASCCP) have included HPV testing for the management of atypical glandular cytology and for the follow-up after treatment for cervical intraepithelial neoplasia. In our study, 96 % of patients were HR-HPV-positive before the initial LEEP. As we did not perform HR-HPV testing before the second LEEP and before hysterectomy in these two groups, we were unable to compare HPV load before and after the initial LEEP treatment.

The treatment options for patients with HSIL and positive margins depend on different factors. The ASCCP updated its consensus guidelines in 2013, as follows: “If CIN2, 3 is identified at the margins of an excisional procedure or post-procedure ECC, cytology and ECC at 4–6 month is preferred, but repeat excision is acceptable and hysterectomy is acceptable if re-excision is not feasible”. The results presented herein may assist in the personalization of the choice of procedure for patients who have HSIL-involved margins after LEEP. It should be considered that the majority of patients with HSIL-involved margins remain disease-free throughout the follow-up period. Therefore, disease management should be individualized based on desired fertility, age, patient preference and other factors. If patients choose to undergo additional treatment, the risk of complications must be weighed against the desire to eradicate potential residual disease.

In our study, the rates of HSIL persistence/recurrence in patients who had a subsequent LEEP and hysterectomy were 31.82 % (7/22) and 20.90 % (14/67), respectively, while that in patients who were selected for close follow-up (TCT or TCT combined with colposcopic-guided biopsy) was 4.02 % (6/149). The mean age of the patients who underwent a subsequent hysterectomy was 47.87 ± 8.71 years, which was significantly higher than the mean age of patients in the other three groups. Significantly more patients who were older than 35 years experienced persistence/recurrence compared with patients who were younger than 35 years (14 % vs. 3 %). Thus, we considered that the presence of HSIL margins is still an important factor associated with recurrence but that this does not always warrant surgical retreatment. A closer, strict surveillance after an initial LEEP is acceptable for younger patients. For patients who are older than 35 years, a subsequent LEEP or a carefully decided hysterectomy is reasonable.

## Conclusion

In patients with HSIL and HSIL-involved margins after an initial LEEP, age is a strong independent predictor of persistence/recurrence. Follow-up with screening cytology and/or biopsy may be considered in younger patients, whereas a secondary LEEP/hysterectomy may be considered in older patients.

One weakness of our study is its retrospective nature and lack of HPV tests before patients received a direct second LEEP and hysterectomy, which, therefore, limits its application. In addition, the follow-up period was relatively short. The strengths of our study are the large size of the study population and the extensive investigation of preoperative variables. We are currently in the process of developing a prospective study to confirm or refute our results.

## Abbreviations

HSIL: High-grade squamous intraepithelial lesion
LEEP: Loop electrosurgical excision procedure
LSIL: Low-grade squamous intraepithelial lesion
CIN: Cervical intraepithelial neoplasia
ECC: Endocervical curettage
HPV: Human papilloma virus
OR: Odd ratio
CI: Confidence interval
ASCCP: American Society for Colposcopy and Cervical Pathology
